# Monthly Summary

**Published:** 1897-04

**Authors:** 


### Monthly Summary.

Nodule on Apex of Root.—A lady, aged about thirty, presented for treatment of a chronic abscess of right superior lateral incisor. Upon examination with a probe it was found that the labial wall of bone at the apex of the root had been destroyed. With the intention of scraping the apex and breaking up the sac, a small right-angled scoop was introduced and passed easily over and around the apex. Upon with-withdrawing it a small nodule of dentine or enamel about the size of an ordinary pinhead was brought away. The abscess was treated with pyrozone four times at intervals of three days when the discharge ceased. Evidently the pulp had been dead for a long time, and the canal filled, as there were large mesial and distal fillings. The case was lost sight of for a year, and in the meantime she had this lateral, and also the right central, which was badly carious, extracted, and is now wearing an artificial denture.

The special interest attached to the case is the fact of finding a nodule at the *apex* of a root, especially of a *single-rooted* tooth.

Enamel nodules are small exerescences, apparently consisting of enamel, occasionally met with upon the roots of teeth. They are generally found upon multiple-rooted teeth situated a little below the neck and often at the junction of the roots. On section they are found to consist of a cone of dentine covered with a rather thick layer of enamel, and often connected with the crow by enamel. Wedl says that "these nodules are the result of localized continuations of the development of the enamel between the enamel already developed basal portion of the roots, and are produced by the strip of the enamel organ which has persisted longer than the rest." Smale shows the cut of one of these nodules on the apex of a *molar* root, and says they may be accounted for by a budding from the tissues concerned in the process of the formation of the tooth. Tomes shows a cut of a nodule situated on the *neck* of a *single-rooted* superior cuspid.—H. L. AMBLER, M. D., D. D. S., in *Ohio Dental Journal*.

---

CREOSOTE possesses undoubted power to relieve the fœter of the expectoration in foul-smelling cases of beonchiectasis and phthisical cavities. In small doses (one to two minims thrice daily) it promotes appetite and tends to stimulate the powers of digestion. Beyond this it is not found that it modifies in an appreciable manner the ordinary course of phthisis.—*Medical Record*.

